# Lipophilic components of diesel exhaust particles induce pro-inflammatory responses in human endothelial cells through AhR dependent pathway(s)

**DOI:** 10.1186/s12989-018-0257-1

**Published:** 2018-05-11

**Authors:** Bendik C. Brinchmann, Tonje Skuland, Mia H. Rambøl, Krisztina Szoke, Jan E. Brinchmann, Arno C. Gutleb, Elisa Moschini, Alena Kubátová, Klara Kukowski, Eric Le Ferrec, Dominique Lagadic-Gossmann, Per E. Schwarze, Marit Låg, Magne Refsnes, Johan Øvrevik, Jørn A. Holme

**Affiliations:** 1 Department of Air Pollution and Noise, Domain of Infection Control, Environment and Health, Norwegian Institute of Public Health, PO Box 4404, Nydalen, N-0403 Oslo, Norway; 2 Division of Laboratory Medicine, Faculty of Medicine, University of Oslo, Oslo, Norway; 3 Norwegian Center for Stem Cell Research, Department of Immunology, Oslo University Hospital, Oslo, Norway; 4 Luxembourg Institute of Science and Technology (LIST), Environmental Research and Innovation (ERIN) Department, Belvaux, Grand Duchy of Luxembourg; 5 Department of Chemistry, University of North Dakota, Grand Forks, ND USA; 6 Inserm U1085, Institut de Recherche en Santé, Environnement, Travail (IRSET), Rennes, France; 7 Université de Rennes 1, Faculté des Sciences pharmaceutiques et biologiques, Rennes, France

**Keywords:** Air pollution, Diesel exhaust particles, Inflammation, Cardiovascular disease, Atherosclerosis, Endothelial cells, 3D tri-culture, Organic compounds, Aryl hydrocarbon receptor

## Abstract

**Background:**

Exposure to traffic-derived particulate matter (PM), such as diesel exhaust particles (DEP), is a leading environmental cause of cardiovascular disease (CVD), and may contribute to endothelial dysfunction and development of atherosclerosis. It is still debated how DEP and other inhaled PM can contribute to CVD. However, organic chemicals (OC) adhered to the particle surface, are considered central to many of the biological effects. In the present study, we have explored the ability of OC from DEP to reach the endothelium and trigger pro-inflammatory reactions, a central step on the path to atherosclerosis.

**Results:**

Exposure-relevant concentrations of DEP (0.12 μg/cm^2^) applied on the epithelial side of an alveolar 3D tri-culture, rapidly induced pro-inflammatory and aryl hydrocarbon receptor (AhR)-regulated genes in the basolateral endothelial cells. These effects seem to be due to soluble lipophilic constituents rather than particle translocation. Extractable organic material of DEP (DEP-EOM) was next fractionated with increasing polarity, chemically characterized, and examined for direct effects on pro-inflammatory and AhR-regulated genes in human microvascular endothelial (HMEC-1) cells and primary human endothelial cells (PHEC) from four healthy donors. Exposure-relevant concentrations of lipophilic DEP-EOM (0.15 μg/cm^2^) induced low to moderate increases in IL-1α, IL-1β, COX2 and MMP-1 gene expression, and the MMP-1 secretion was increased. By contrast, the more polar EOM had negligible effects, even at higher concentrations. Use of pharmacological inhibitors indicated that AhR and protease-activated receptor-2 (PAR-2) were central in regulation of EOM-induced gene expression. Some effects also seemed to be attributed to redox-responses, at least at the highest exposure concentrations tested. Although the most lipophilic EOM, that contained the majority of PAHs and aliphatics, had the clearest low-concentration effects, there was no straight-forward link between chemical composition and biological effects.

**Conclusion:**

Lipophilic and semi-lipophilic chemicals seemed to detach from DEP, translocate through alveolar epithelial cells and trigger pro-inflammatory reactions in endothelial cells at exposure-relevant concentrations. These effects appeared to be triggered by AhR agonists, and involve PAR-2 signaling.

**Electronic supplementary material:**

The online version of this article (10.1186/s12989-018-0257-1) contains supplementary material, which is available to authorized users.

## Background

More than 90% of the world’s population live in areas with unhealthy air according to WHO [1]. Particulate matter (PM), especially fine PM (PM_2.5_), is a leading environmental cause of cardiovascular disease (CVD) [2–5], and has been linked to development and exacerbation of endothelial dysfunction and atherosclerosis in a number of experimental and epidemiological studies [6–8]. Atherosclerosis is initiated by endothelial dysfunction and can lead to myocardial infarction, cerebrovascular and peripheral vascular disease [9], making it the major cause of deaths due to CVD [10]. It is an inflammatory disorder of the arteries, a process that involves oxidative stress, increased endothelial permeability, leukocyte adhesion and other inflammatory reactions [11].

Diesel engines are major contributors to PM_2.5_ in urban environments [12, 13]. Thus, diesel exhaust particles (DEP) have frequently been used as a model to explore the mechanisms of PM-induced CVD [14, 15]. Much of the biological effects of DEP, including pro-inflammatory responses, have been attributed to soluble organic chemicals (OC) adherent to the carbon core of the particles [16–19]. How DEP and other inhaled PM can cause adverse effects in the endothelium is still debated despite extensive research. One common theory is that PM may cause pulmonary macrophages and epithelial cells to release pro-inflammatory mediators into the circulation, leading to systemic effects [20]. However, a recent review concluded that neither pulmonary nor systemic inflammation is a prerequisite for PM-induced atherosclerosis or endothelial dysfunction [14]. An alternative explanation is that PM_2.5_ and its constituents could affect endothelial cells more directly. Recently, inhaled gold nanoparticles (2-200 nm) were shown to translocate from the lung into the circulation and preferentially accumulated at sites of inflammatory vascular lesions in mice and humans [21]. This suggests that nano-sized combustion particles may also be transported to sites of endothelial injury in a similar way. Thus, DEP translocated into the circulation, may deliver its “organic cargo” directly to endothelial cells. However, studies using model particles rich in polycyclic aromatic hydrocarbons (PAH) suggest that PAHs are released from the particles, passes through the alveolar wall into the circulation, and are distributed systemically [22–24]. This suggests that translocation of DEP and other combustion particles across the alveolar wall may not be necessary for soluble OC to be transferred into the circulation and reach the endothelium, also distant from the lung.

Although pulmonary and systemic inflammation may not be the prime drivers of adverse PM-induced effects on the endothelium [14], inflammatory responses have nevertheless a key role in endothelial dysfunction and atherosclerosis. Pro-inflammatory mediators such as cytokines, chemokines and matrix metalloproteinases (MMPs) are crucial in the different developmental stages of the disease, and endothelial cells appear to orchestrate these events [11, 25]. In line with this, inflammation and oxidative stress in the arterial wall seems consistently associated with PM-induced vasomotor dysfunction and plaque progression [14]. Thus, local rather than systemic inflammation may be a prerequisite for development of endothelial dysfunction by PM-exposure.

DEP appear to cause cellular effects through multiple mechanisms, and the pro-inflammatory effects most likely arise from the combined activation of several pathways [26, 27]. It is known that PAHs and other OC from DEP can bind the aryl hydrocarbon receptor (AhR), which in turn may lead to an increased expression of genes linked to inflammation and xenobiotic metabolism [28]. AhR may regulate inflammation through non-genomic signaling, cross-talk with transcription factors such as the nuclear factor-κB (NF-κB), and some cytokines also contain AhR-response elements in their promotor region [29, 30]. Furthermore, metabolism of OC from DEP by various cytochrome P450 (CYP) enzymes may form reactive oxygen species (ROS) and reactive electrophilic metabolites [31] with potential to trigger inflammation. In addition, recent studies suggest that the protease activated receptor-2 (PAR-2), a G-protein coupled receptor, regulates matrix metalloproteinase-1 (MMP-1) and interleukin-6 (IL-6) in human bronchial epithelial cells exposed to DEP and DEP-EOM [17, 32]. PARs are also constitutively expressed in the vascular endothelium where they regulate tone, permeability and coagulation as well as inflammation [33, 34]. Thus, AhR and PAR-2 as well as redox-regulated responses could likely be involved in the effects of DEP and OC from DEP in endothelial cells.

In the present study we have explored potential mechanisms involved in PM-induced endothelial inflammation by various in vitro models, using DEP as a surrogate for traffic-derived PM. We asked: i) does DEP affect endothelial cells via OC, ii) which classes of chemicals in DEP-OC are inducing inflammatory reactions, and iii) which cellular mechanisms are involved. The use of exposure-relevant concentrations/doses in vitro are important to ensure that the mechanisms explored could be relevant for adverse effects in real-life [35]. Based on in vivo calculations for high-risk individuals exposed to PM levels of 79 μg/m^3^, Li and coworkers estimated PM_2.5_ deposition rates over 24 h to be 204 μg/cm^2^ in the nasopharyngeal, 2.3 μg/cm^2^ in the tracheobronchial and 0.05 μg/cm^2^ in the alveolar regions [36]. Acknowledging that our in vitro systems were bolus-exposed, an additional central focus was to explore effects at these concentrations. We found that DEP applied on the apical surface of alveolar cells in a 3D tri-culture model of the alveolar-capillary barrier, induced expression of pro-inflammatory genes and markers of AhR-signaling in the basolateral endothelial cells at concentrations down to exposure-relevant levels. These responses appeared not to depend on particle translocation. By exposing endothelial cell monocultures (HMEC-1 and primary human endothelial cells; PHEC) to fractionated extractable organic material of DEP (DEP-EOM), we confirmed that DEP-EOM induced inflammation-associated genes through mechanisms partly depending on AhR, PAR-2 and redox responses. At the lowest, most exposure-relevant concentrations, the most lipophilic DEP-EOM seemed to have the strongest effects on the expression of pro-inflammatory genes in HMEC-1 and PHEC. This DEP-EOM fraction also had the highest content of semi-volatile OC, especially PAHs and aliphatic hydrocarbons.

## Results

### DEP-induced gene expression in 3D tri-culture model

**Fig. 1:**
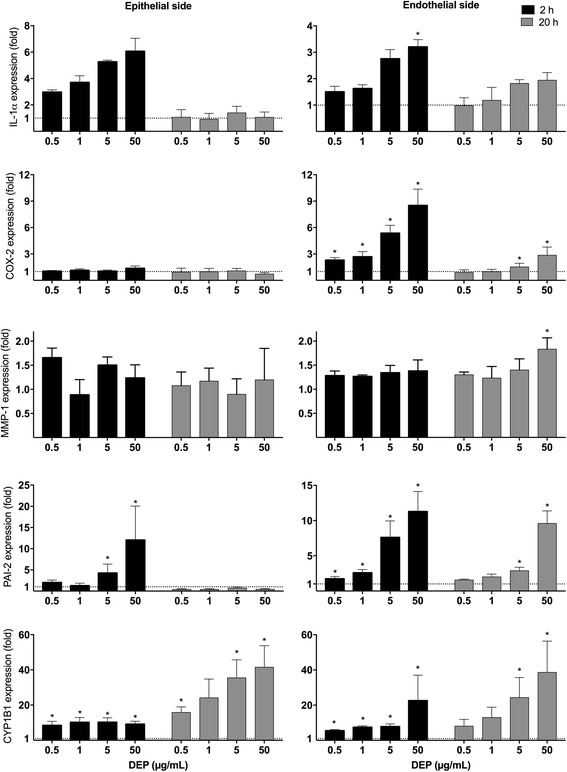
Effect of DEP exposure on gene expression in a 3D tri-culture model. Increasing concentrations of DEP were applied to the epithelial side of the 3D tri-culture. After 2 and 20 h of exposure, alveolar and endothelial cells were harvested and the expressions of IL-1α, COX-2, MMP-1, PAI-2 (SERPINB2) and CYP1B1 mRNAs were measured by q-PCR. The mRNA levels are presented relative to gene expression in cells exposed to DMSO, represented by the dotted line at 1. The results are expressed as mean ± SEM (*n* = 3). *Statistically significant difference from unexposed controls

We hypothesized that OC from DEP could translocate through alveolar epithelial cells and reach endothelial cells. To test this we utilized an established 3D tri-culture composed of alveolar type-II A549 cells and macrophage-differentiated THP-1 cells on the apical (epithelial) side of a microporous membrane and endothelial cells (EA.hy926) on the basolateral side [37, 38]. We define the model as a 3D tri-culture model of the alveolar-capillary barrier based on the 3D interaction between the various cells at both sides of the insert as described by Klein et al. [38, 39]. This 3D tri-culture model was exposed to different concentrations of DEP at the epithelial side for 2 and 20 h. The DEP that previously have been characterized by Totlandsdal et al. [40], rapidly induced pro-inflammatory and AhR-regulated genes in cells on both the epithelial side and the endothelial side of the 3D tri-culture (Fig. 1). More specifically, IL-1α (2-5 fold), PAI-2 (3-15 fold) and CYP1B1 (8-40 fold) mRNA expressions were up-regulated in cells on the epithelial side. The up-regulations observed in EA.hy926 cells were in general similar to those on the epithelial side. However, DEP induced COX-2 expression in EA.hy926 (3-10 fold), in the absence of any apparent response on the epithelial side. This increase was statistically significant already after 2 h exposure to the lowest concentration tested (0.5 μg/mL or 0.12 μg/cm^2^). At the highest concentration, DEP also induced a 2-fold increase in MMP-1 expression in the endothelial cells, whereas cells on the epithelial side did not up-regulate MMP-1 (Fig. 1). In contrast, SiO_2_ nanoparticles (SiNP) used as control particles without soluble OC only activated gene expression at the epithelial side and not in the endothelial cells of the tri-culture (Additional file 1: Figure S1A), despite being able to trigger a substantial COX-2 increase when exposed directly to EA.hy926 cells in monoculture (Additional file 1: Figure S1B).This suggests that nanoparticles did not reach the endothelial cells through the layer of epithelial cells in the tri-culture, at least not in sufficient quantities to trigger a response. The most likely interpretation of these findings is that particles were not translocated into the endothelial layer of the 3D model, and that the endothelial responses to DEP-exposure were rather due to soluble OCs passing through the epithelial layer.

### Chemical characterization of various DEP-EOM

**Fig. 2:**
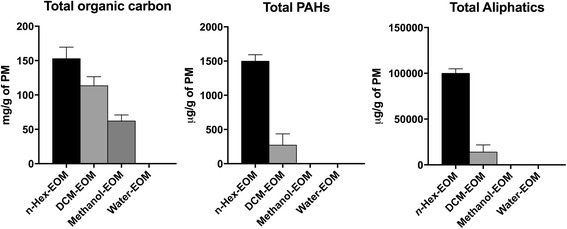
Characterization of DEP-EOM. The soluble organic carbon/chemicals were extracted directly from the native particulates by sequential washing with n-hexane, DCM and methanol at 100 °C (under pressure), followed by a final washing with water at 25 °C. Total content of organic carbon was analyzed by thermal optical analysis, while content of PAHs and aliphatics were measured by GC-FID/MIS, as described under materials and methods. The extraction was done in three parallels and the results are expressed as mean ± SEM (*n* = 3)

The above results support a major role of soluble OC in the effects of DEP. We therefore extracted organic material from DEP by sequential washing under pressure at 100 °C, with solvents of increasing polarity*: n*-hexane, dichloromethane (DCM), and methanol, followed by a final washing with water at 25 °C. The chemical compositions of these four fractions, from here on referred to as *n-*Hex-EOM, DCM-EOM, Methanol-EOM and Water-EOM, were analyzed for total content of carbon, amount of PAHs (and their derivatives) and aliphatic hydrocarbons (Fig. 2). In line with previous analyses, the relative amount of organic versus elemental carbon in this DEP was approximately 60 and 10%, respectively [41]. As expected for DEP [41], we found that most of the OC was extracted with *n*-hexane and DCM, with remaining 19% recovered in the methanol extract. OC was not detected in the water extract. The most lipophilic *n*-hexane extract contained almost 90% of the PAHs and aliphatic hydrocarbons, while the rest was obtained with DCM (see Additional file 1: Table S1, for an overview of detected species). The relative amount of different PAH species corresponded to previous analyses [41] with phenanthrene (and methylated phenanthrene or anthracene), fluoranthene, pyrene, chrysene, xanthone and 1-nitropyrene being the most abundant species detected (data not shown). Notably, the amount of volatile/semi-volatile compounds extracted also decreased according to polarity of the solvents (Additional file 1: Figure S2).

### DEP-EOM-induced gene-expression in HMEC-1

The observed effects on endothelial cells in the 3D tri-culture exposed to a DEP rich in OC, combined with the previously reported marginal effects on endothelial cells in a comparable 3D tetra-culture exposed to DEP (SRM2975) with little OC, strongly suggests an important role of OC from DEP. Although the extracts not necessarily reflects the specific compounds reaching the endothelial cells of the 3D tri-culture, they contain a mixture of OC similar to what has been reported in other studies on DEP [41, 42]. We next investigated if endothelial cells exposed directly to DEP-EOM responded in a similar manner as the endothelial cells exposed indirectly to DEP through the epithelial cells in the 3D tri-culture. As the EA.hy926 cell line used in the 3D tri-culture is a endothelial/epithelial hybrid [43], we chose another well described endothelial cell line for the next experiments: the HMEC-1 cells which are of microvascular origin [44].

**Fig. 3:**
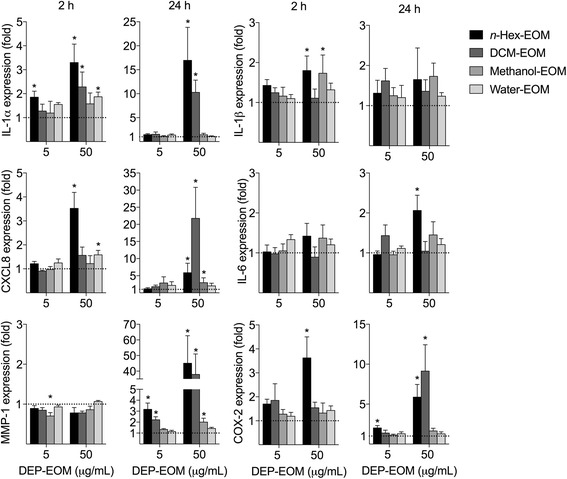
Effects of DEP-EOMs on expression of pro-inflammatory genes in HMEC-1 cells. Cells were exposed to DEP-EOMs at concentrations corresponding to 5 and 50 μg/mL (0.75 and 7.5 μg/cm^2^) of native particles, or vehicle (DMSO) alone for 2 and 24 h. The expressions of IL-1α, IL-1β, IL-6, CXCL8, COX-2 and MMP-1 were measured by q-PCR. The mRNA levels are presented relative to gene expression in cells exposed to DMSO, represented by the dotted line at 1. The results are expressed as mean ± SEM (*n* = 4). *Statistically significant difference from unexposed controls

**Fig. 4:**
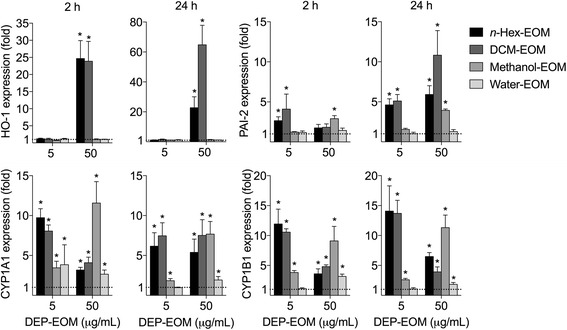
Effects of DEP-EOMs on expression of HO-1 and AhR-regulated genes in HMEC-1 cells. Cells were exposed to DEP-EOMs at concentrations corresponding to 5 and 50 μg/mL (0.75 and 7.5 μg/cm^2^) of native particles, or vehicle (DMSO) alone for 2 and 24 h. The expressions of CYP1A1, CYP1B1, PAI-2 and HO-1 were measured by q-PCR. The mRNA levels are presented relative to gene expression in cells exposed to DMSO, represented by the dotted line at 1. The results are expressed as mean ± SEM (*n* = 4). *Statistically significant difference from unexposed controls

HMEC-1 were exposed to DEP-EOM concentrations of 5 and 50 μg/mL corresponding to 0.75 and 7.5 μg/cm^2^ of originally unwashed DEP. These concentrations did not appear to be cytotoxic, as initially screened by the WST-1 proliferation assay and judged visually by microscopy (Additional file 1: Figure S3). IL-1 α/β, IL-6, CXCL8, MMP-1 and COX-2 mRNA concentrations were measured to explore pro-inflammatory effects. ROS-related effects were addressed by assessing HO-1 expression and AhR activity by measuring the AhR response genes CYP1A1, -1B1 and PAI-2. The lipophilic DEP-EOM fractions, particularly at the highest concentration, increased the expression of the pro-inflammatory genes measured (Fig. 3). The effects on CXCL8 expression and lack of effect on IL-6 expression was further confirmed by ELISA, showing that CXCL8 protein (but not IL-6) was secreted by HMEC-1 cells upon 24 h exposure to the *n*-Hex- and DCM-EOM (50 μg/mL), but not by the methanol and water extracts (Additional file 1: Figure S4). Most interestingly, expression of IL-1α, COX-2, and MMP-1 was increased (2-3 fold) at the low concentration of the *n-*hexane extract, corresponding to 5 μg/mL (0.75 μg/cm^2^) of original DEP (Fig. 3). In addition, the low concentration of the DCM extract also induced an increase in MMP-1 (2-fold) after 24 h (Fig. 3). The expression of the AhR-response genes CYP1A1 and CYP1B1 was induced by both concentrations of all the fractions, but *n-*hexane- and DCM-EOM had more marked effects at the lowest concentration (Fig. 4). Previous studies with this specific DEP have shown that CYP1A1-responses in bronchial epithelial BEAS-2B cells peaked at 4 μg/cm^2^, and were reduced at higher concentrations [40]. In line with this, the CYP1A1 and -1B1 responses induced by the two most potent DEP-EOM fractions (*n-*hexane and DCM) also appeared to be reduced at the highest concentration (Fig. 4). The AhR response-gene PAI-2 [45] was induced by all fractions, except the water extract. Similar to the CYP- responses, PAI-2 was more strongly increased by the *n-*Hex- and DCM-EOM, at least at low concentrations. Furthermore, HO-1 was only upregulated at 50 μg/mL and not at 5 μg/mL, indicating that responses observed at the lowest concentration were triggered in the absence of measured oxidative stress.

The above results suggest that pro-inflammatory gene expression was predominately affected by the two most lipophilic DEP-EOM fractions: *n-*Hex- and DCM-EOM. At the lowest concentration, effects were the most pronounced for the *n-*hexane extract.

### DEP-EOM-induced gene-expression in PHEC

The responses of cell lines used in our systems are not representative for real exposure. We thus further explored the relevance of our findings by using primary human endothelial cells (PHEC) obtained from adipose tissue of four healthy donors [46]. These PHEC were of high purity, as indicated by 99% CD31 positive cells (Additional file 1: Figure S5). The cells were exposed to low concentrations of the different DEP-EOM fractions, corresponding to 1 and 5 μg/mL (0.15 and 0.75 μg/cm^2^) of native particles, for 24 h. No visual cytotoxicity was observed in cells exposed to either of the DEP-EOM fractions at the highest concentration, as exemplified with *n-*Hex-EOM (Additional file 1: Figure S3B).

**Fig. 5:**
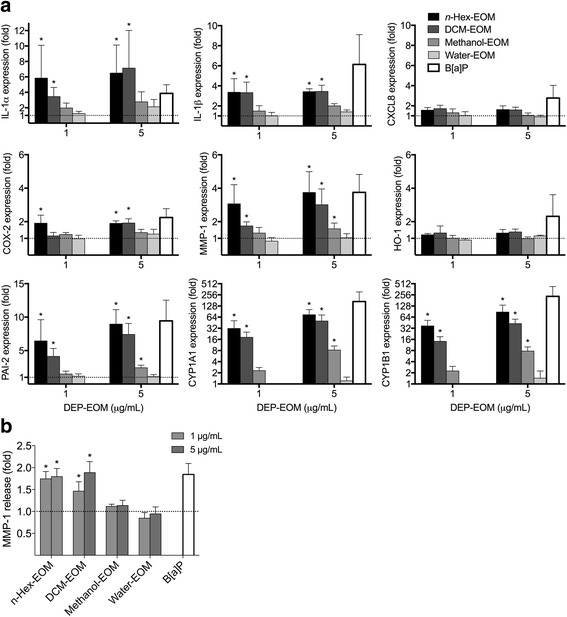
Effects of lipophilic DEP-EOMs in PHEC. PHEC were exposed to DEP-EOM at concentrations corresponding to 1 or 5 μg/mL (0.15 and 0.75 μg/cm^2^) of native particles, vehicle (DMSO) or 1 μM B[*a*]P (positive control) for 24 h. The expressions of IL-1α, IL-1β, CXCL8, COX2, MMP-1, HO-1, CYP1A1, CYP1B1 and PAI-2 was measured by q-PCR (**a**). MMP-1 up-regulation were confirmed with ELISA, showing 45-90% higher levels of MMP-1 in growth medium from PHEC exposed to *n*-hexane or DCM (**b**). The mRNA levels are presented relative to gene expression in cells exposed to DMSO, represented by the dotted line at 1. Data are based on results from experiments with PHEC from 4 healthy donors. The results are expressed as mean ± SEM (A/B: *n* = 4). *Statistically significant difference from unexposed controls

As in HMEC-1, the lipophilic DEP-EOM fractions induced inflammation-associated genes, as well as CYP1A1 and CYP1B1 in PHEC, while the hydrophilic extracts had negligible effects (Fig. 5a). Most notably lipophilic DEP-EOM caused statistically significant up-regulation (2-7 fold) of IL-1α, IL-1β, COX-2 and MMP-1 expression, even at the lowest concentration (1 μg/mL, corresponding to 0.15 μg/cm^2^ of native particles), while CXCL8 and HO-1 were unaffected after 24 h exposure. Furthermore, CYP1A1, CYP1B1 and PAI-2 were induced by all DEP-EOM fractions, except the water extract (Fig. 5a), and CYP1A1 and CYP1B1 mRNAs were more strongly induced in PHEC (15-90 fold), than in HMEC-1. Of interest, the MMP-1 mRNA up-regulation was confirmed with ELISA; MMP-1 protein levels were increased by 45-90% in PHEC exposed to *n-*Hex- or DCM-EOM (Fig. 5b).

### Mechanisms of DEP-EOM-induced gene expression in HMEC-1

**Fig. 6:**
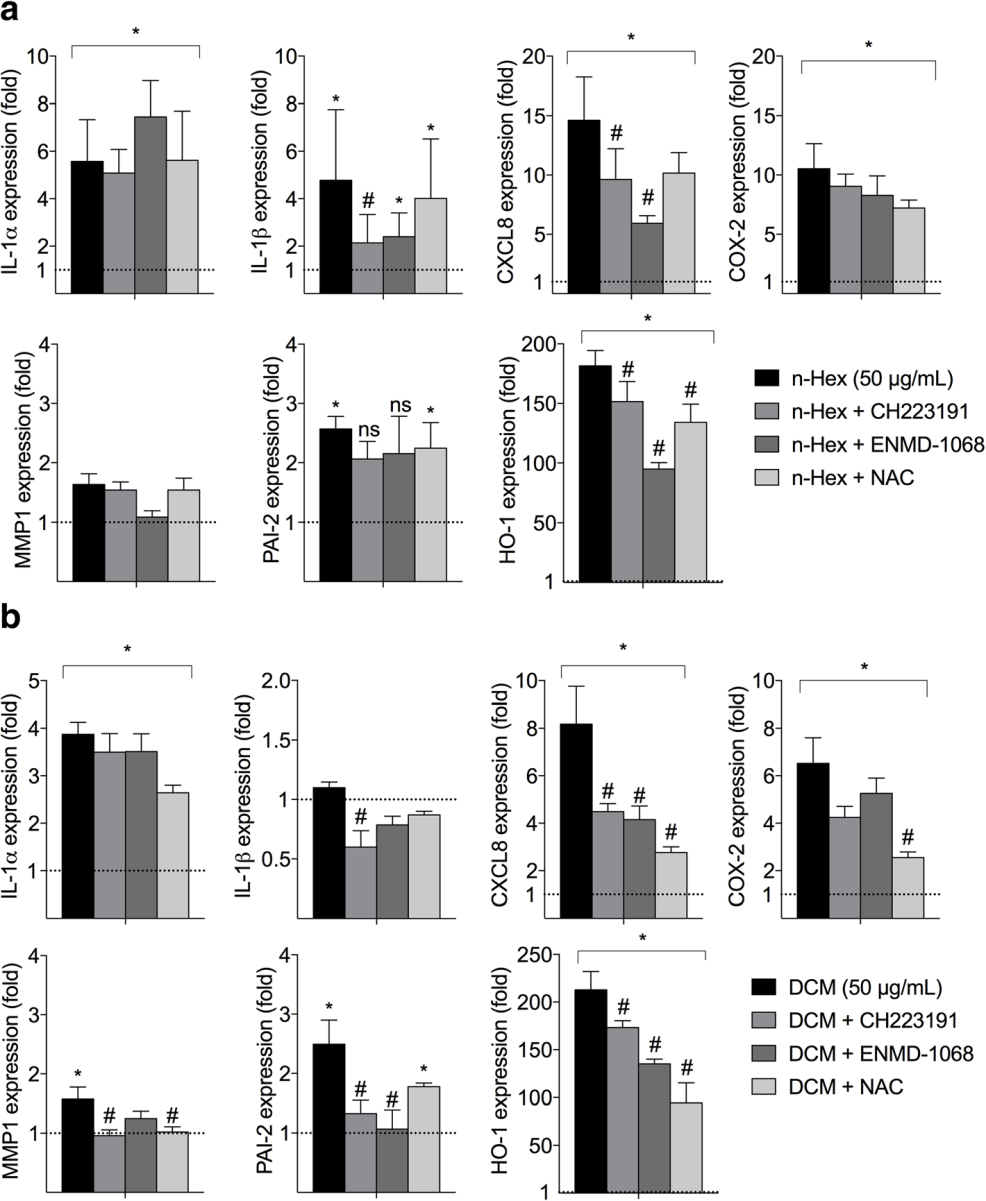
Effects of inhibitors on DEP-EOM-induced gene expression in HMEC-1. Cells were pre-treated with the anti-oxidant NAC (2 mM), the AhR inhibitor CH223191 (1.0 μM), or the PAR-2 inhibitor ENMD-1068 (2.5 mM) for 30 min prior to exposure to the lipophilic *n*-Hexane- (**a**) or DCM-soluble (**b**) fractions of DEP-EOM at a concentration corresponding to 50 μg/mL (7.5 μg/cm^2^) of native particles, or vehicle (DMSO) alone, for 5 h. Gene expression was measured by q-PCR. The mRNA levels are presented relative to gene expression in cells exposed to DMSO, represented by the dotted line at 1. The results are expressed as mean ± SEM (A/B: *n* = 3). *Statistically significant difference from unexposed controls. #Statistically significant difference from cells exposed to DEP-EOM

To elucidate the mechanisms involved in the regulation of the pro-inflammatory responses induced by *n-*Hex- and DCM-EOM (50 μg/mL), we pretreated HMEC-1 cells with pharmacological inhibitors targeting AhR (CH223191; 1.0 μM) or PAR-2 (ENMD-1068; 2.5 mM), and to target ROS we used the anti-oxidant N-acetylcysteine (NAC; 2.0 mM). To minimize potential unspecific effects of the inhibitor/antioxidant treatment, these experiments were terminated after 5 h exposure. Generally *n-*Hex-EOM appeared to induce slightly stronger effects on IL-1α, IL-1β, COX-2 and CXCL8 mRNAs, while DCM-EOM had somewhat more effect on HO-1 at this time-point (Fig. 6a). CH223191-treatment attenuated IL-1β expression in *n-*Hex-EOM-exposed cells. CXCL8 and HO-1 responses induced by the two lipophilic DEP-EOM fractions were partly reduced by both CH223191 and ENMD-1068. Of interest, ENMD-1068 caused stronger reduction in both *n-*Hex-EOM-induced CXCL8 and HO-1 expression compared to NAC, while the opposite was the case for DCM-EOM-induced effects on these genes. IL-1α, COX-2, MMP-1 and PAI-2 levels induced by *n-*Hex-EOM were not significantly affected by any of the inhibitors (Fig. 6a). By comparison, NAC suppressed COX-2 and MMP-1 expression induced by DCM-EOM and CH223191 suppressed both MMP-1 and PAI-2 (Fig. 6b). Furthermore, DCM-EOM-induced PAI-2 was blocked by ENDM-1068. However, as both MMP1 and PAI-2 were only weakly up-regulated at this time-point, it was difficult to measure any significant effects of inhibitors. Overall, the data suggest that the AhR (CH223191) and PAR-2 (ENMD-1068) are involved in the inflammation-linked responses (IL-1β, CXCL-8 and HO-1) of both *n-*Hex- and DCM-EOM. Moreover, IL-1α expression was not affected by any of the tested inhibitor/antioxidant treatments, which underscores that DEP-induced inflammation could hardly be explained by the few mechanisms explored in the present study.

### Mechanisms of DEP-EOM-induced gene expression in PHEC

**Fig. 7:**
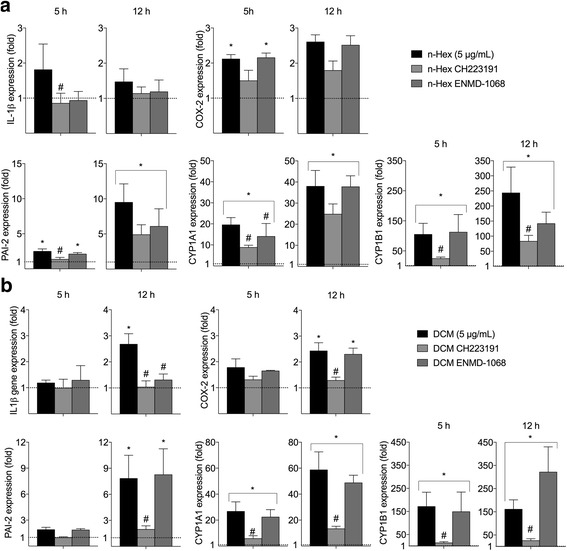
Effects of inhibitors on DEP-EOM-induced gene expression in PHEC. Cells from one donor were pre-treated with the AhR inhibitor CH223191 (1.0 μM), or the PAR-2 inhibitor ENMD-1068 (2.5 mM) for 30 min prior to exposure to the lipophilic *n*-Hexane- (**a**) or DCM-soluble (**b**) fractions of DEP-EOM at a concentration corresponding to 5 μg/mL (0.75 μg/cm^2^) of native particles, or vehicle (DMSO) alone, for 5 h. Gene expression was measured by q-PCR. The mRNA levels are presented relative to gene expression in cells exposed to DMSO, represented by the dotted line at 1. The results are expressed as mean ± SEM (5 h *n* = 3; 12 h *n* = 4). *Statistically significant difference from unexposed controls. #Statistically significant difference from cells exposed to DEP-EOM

Finally, we also explored the mechanisms involved in the regulation of the pro-inflammatory responses induced by *n-*Hex- and DCM-EOM in batches of PHECs from one donor. PHECs were pretreated with the AhR- and PAR-2 inhibitors CH223191 (1.0 μM) and ENMD-1068 (2.5 mM) for 30 min prior to 5 or 12 h exposure with the two lipophilic DEP-EOM fractions at a concentration corresponding to 5 μg/ml (0.75 μg/cm^2^) of original DEP. As no effect was observed on HO-1 expression in PHECs (Fig. 5), effects of NAC were not investigated. Like in HMEC-1 cells, induction of IL-1β by both *n-*Hex- and DCM-EOM was attenuated by CH223191-treatment to near basal levels (Fig. 7). ENMD-1068 also blocked DCM-EOM-induced IL-1β. A similar effect of the PAR-2 inhibitor was also observed for *n-*Hex-EOM-induced IL-1β, but this reduction was not statistically significant. Furthermore, CH223191, but not ENMD-1068 attenuated both COX-2 and PAI-2 responses in the DCM-EOM-exposed PHECs. Notably, COX-2 expression was not significantly reduced by CH223191 in *n-*Hex-EOM-exposed cells, but the statistically significant increase in COX-2 was lost in cells treated with the AhR-inhibitor. As expected, the induction of CYP1A1 and -1B1 expression was primarily suppressed by CH223191. However, some effects of ENMD-1068 were observed on *n-*Hex-EOM-induced CYP1A1 at 5 h. These results suggest that both AhR and PAR-2 signaling are involved in regulation of pro-inflammatory responses for these relatively low-level exposures of lipophilic and semi-lipophilic DEP-EOM fractions.

## Discussion

Recent findings from ApoE −/− mice suggest that atherosclerotic effects of PM_2.5_ is due to semi-volatile OC attached to the particles [47]. In line with this, the present study shows that DEP trigger pro-inflammatory responses in endothelial cells through release of lipophilic OC that could transfer across alveolar epithelial cells. These responses were triggered at concentrations down to exposure-relevant levels and appeared partly dependent on AhR, PAR-2 and (at least at higher concentrations) redox-regulated responses.

In this study we found that DEP-exposure increased expression of IL-1α, PAI-2 and CYP1B1 in cells on the epithelial side of a 3D tri-culture system, in response to concentrations of DEP in the range 0.5 – 50 μg/mL corresponding to 0.12-12 μg/cm^2^. The lowest exposure concentration with marked biological effects in the 3D tri-culture (0.12 μg/cm^2^) and the DEP-equivalent exposure concentration of EOM in PHEC (0.15 μg/cm^2^) are within the range estimated for alveolar deposition [36]. Most importantly, we found a rapid and marked induction of the same genes as well as COX-2 and MMP-1 in endothelial cells located on the basolateral side. The AhR marker genes PAI-2 and CYP1B1 [28, 45] were also increased in the endothelial cells, indicating that organic compounds of DEP containing AhR-agonists reached the endothelial cells after exposure of the epithelial cells at the apical side. PAI-2 is seemingly regulated via AhR non-canonical signaling [45], and its upregulation indicates that additional pathways to the AhR-ARNT pathway is activated. Of notice, results obtained by the use of 10 nm silica-nanoparticles at fairly high concentrations (SiNP, used as reactive control-particles without soluble constituents), suggest that DEP-induced effects in the basolateral endothelial cell layer were not due to translocation of particles from the apical compartment, nor to release of pro-inflammatory mediators by the alveolar epithelial cells. Thus, organic compounds most likely detach from DEP and translocate trough the epithelial layer and into the endothelial cells, triggering inflammatory reactions in the microvasculature. A recent study exposing a comparable tetra-culture system of the alveolar-capillary barrier to the standard DEP NIST SRM2975 did not result in any effects on pro-inflammatory genes, and only modest increases in CYP1A1 and Nrf2 in the basolateral endothelial cells [38]. This lack of effect is most likely due to the very low OC-concentration (2%) in SRM 2975, which thus corroborates our suggestion of a central role of soluble OC in the inflammogenic effects from this DEP and other more OC-rich DEPs. Earlier findings using PAH-rich butadiene soot, have shown that PAHs are transferred from the particle surface to the cell membrane and enters the cytosol of lung epithelial cells, without particle uptake [22]. Based on these findings and our present results we suggest that lipophilic chemicals from DEP deposited in the alveoli, could reach the microvascular endothelium, by transfer through the lipid-rich membranes of alveolar epithelial cells. Moreover, in vivo studies with PAH-coated carbon particles have shown that much of the PAHs are rapidly transferred into the circulation in an un-metabolized state [23], and likely transported by low density lipoproteins from the lungs to cells of the artery wall [48–50]. In fact, PAH-adducts have been detected in atherosclerotic plaques, and PAH-exposure induce endothelial inflammation and progression of atherogenesis [51, 52].

The majority of the observed effects of DEP appeared to be due to lipophilic and semi-lipophilic compounds extractable by *n*-hexane and DCM. These two DEP-EOM also contained most of the extractable OC. The highest concentrations of the methanol fraction, containing more water-soluble compounds, induced PAI-2, CYP1A1, CYP1B1 and cytokines slightly. This could be caused by small amounts of lipophilic OC left by the two preceding extractions. On the other hand, hydrophilic OC from DEP has been reported to affect CYP-expression [53]. Nevertheless, the two hydrophilic extracts had negligible effects on expressions of inflammation-associated genes and CYP-enzymes in HMEC-1 and PHEC, compared to the two more lipophilic extracts. This is in line with previous findings by us and others, showing that lipophilic extracts from DEP induced CXCL8 and IL-6 responses in human bronchial BEAS-2B cells [18, 32]. Interestingly, the majority of volatile/semi-volatile compounds were present in *n*-Hex- and DMC-EOM, in accordance with the findings of Keebaugh et al. [47].

Chronic or repeated low-grade inflammation in the endothelium is considered to contribute to development and exacerbation of endothelial dysfunction and atherosclerosis [11, 14, 25, 54]. In line with this, our results show that soluble organic material from DEP may trigger low to moderate increases in several pro-inflammatory genes in the endothelial cells. The important role of IL-1 cytokines in the development of CVD is highlighted by ongoing medical trials investigating IL-1β as therapeutic target in treatment of CVD [55, 56]. In the 3D tri-culture, HMEC-1 and PHEC, IL-1 cytokines were induced by DEP or DEP-EOM. Of special interest, in PHEC we observed marked effects on IL-1α and IL-1β mRNA expression (3-6 fold) even at the lowest concentration of exposure. COX-2 was the most sensitive inflammation-associated gene in the 3D tri-culture, HMEC-1 and PHEC. COX-2 is present in inflamed vessels and highly expressed in atherosclerotic lesions; where it can potentially produce large amounts of prostanoids and PGE2. PGE2 can promote the expression of MMPs leading to tissue destruction and destabilization of atherosclerotic plaques [57, 58]. Notably, MMP-1 was induced in HMEC-1 and PHEC as well as the 3D tri-culture after exposure for 20 h or more. It is thus tempting to speculate that the induction and release of MMP-1 at 24 h, was caused at least partly through COX-2-mediated PGE2 production. Taken together, DEP induced increased endothelial expression of pro-inflammatory genes considered relevant in development and progression of atherosclerosis.

DEP most likely initiates cellular responses through a number of different constituents and multiple triggering mechanisms, and pro-inflammatory effects arise from the combined activation of several pathways. Generally, particles and soluble particle components can trigger inflammatory responses through four central events: (i) formation of ROS, (ii) interaction with the lipid layer of cellular membranes, (iii) activation of receptors, ion channels and transporters on the cell surface, and (iv) interaction with intracellular molecular targets including receptors [26, 59]. In this study we have addressed three of these triggering events: ROS formation, activation of membrane receptors (PAR-2) and the intracellular receptor AhR. These mechanisms are of interest since they have been implicated in the pathogenesis of CVD, most notably through their effects on pro-inflammatory signaling [34, 60–63]. Of notice, the relative impact of inhibitor- and antioxidant-treatment varied considerably with both the type of exposure (*n-*Hex- or DCM-EOM) and the genes investigated. This suggests that the two lipophilic extracts induce effects at least partly by different constituents and through different pathways, which is consistent with the notion that a number of mechanisms are involved in the effects from complex exposures such as DEP.

AhR is a ligand-activated transcription factor with affinity for planar aromatic compounds including several PAHs and dioxins, and is among the most well studied xenobiotic receptors [28]. The vasculature is suggested to be a target of PAH exposure [64–66], and AhR-ligands have been shown to disrupt endothelial function, causing atherosclerosis, [60, 62]. In the present study AhR seemed to be partly involved in the regulation of DEP-EOM-induced IL-1β, CXCL8, MMP-1 and HO-1 in HMEC-1. In PHEC the role of AhR in mediating effects of *n-*hexane- and DCM-EOM was similar, CH223191 reduced IL-1β and COX-2. This effect did not seem to be cell-line dependent. AhR could potentially regulate pro-inflammatory genes such as IL-1β and CXCL-8 through cross-talk with the nuclear factor-κB (NF-κB) family of transcription factors or through binding to AhR-response elements (XREs) in their promoter region [29, 30, 67]. However, the effect of AhR-inhibition on COX-2 expression, could also be related to non-genomic signaling [68]. Furthermore, it has been found that AhR ligands induce MMP-1 in human bronchial cells [69], the observed effects on MMP-1 could thus be related to AhR activation. Most importantly, these results indicate that AhR-ligands contribute to the pro-inflammatory effects from lipophilic DEP-EOM in endothelial cells.

G-coupled receptors including PAR are central in endothelial inflammatory responses [33, 34], and PAR-2 has been reported to regulate MMP-1 in bronchial epithelial cells exposed to DEP [17]. Inhibiting PAR-2 with ENMD-1068 caused marked reductions of CXCL8, HO-1 and PAI-2 in HMEC-1. Interestingly, PAR-2 inhibition reduced the effect of the *n-*hexane extract on CXCL8 and HO-1 more markedly than NAC. Thus CXCL8, HO-1 and PAI-2 expression in HMEC-1 seem to be linked to PAR-2-regulated pathways. In PHEC it seemed that PAR-2 partially regulated IL-1β and CYP1A1 (Fig. 6). We have previously found that DEP-induced IL-6 was reduced by PAR-2 silencing in BEAS-2B [32]. Taken together, it seems that PAR-2 contributes to the signaling pathways mediating inflammatory effects of DEP in both bronchial and endothelial cells. PAR-2 is activated by protease-mediated cleavage of the n-terminal domain [33, 34]. Unless DEP and DEP-EOM contains protease activity, it seems more likely that PAR-2 is not directly targeted by the particles or particle components, but rather trans-activated in response to some upstream triggering mechanism. As both PAR-2 and AhR could initiate calcium signaling [17, 68], and since AhR is known to interact with a number of different cellular pathways [70], it is tempting to speculate that there could be a link between signaling from these two receptors in DEP-exposed cells.

The oxidative stress paradigm has dominated the understanding of how DEP and other particulates trigger inflammatory responses in various cell types [36, 71]. In HMEC-1 only the highest concentrations of *n-*Hex- and DCM-EOM induced HO-1. The DCM extract induced the strongest increase in HO-1 expression, at least at later time-points, and the suppressive effects of NAC was most pronounced in DCM-EOM-exposed cells. This correlates well with previous findings that the mid-polar and non-polar OC of wood smoke PM caused GSH depletion in RAW 264.7 macrophages [72] and the notion that DCM have higher levels of redox reactive OC than *n-*hexane [41]. Of all the genes investigated, CXCL8 expression appeared to be most closely associated with HO-1 expression, indicating a central role of redox-regulation. However, while HO-1, CXCL8 and COX-2 gene expressions were partially reduced by NAC, IL-1α, IL-1β and PAI-2 appeared to be unaffected by the antioxidant treatment. Moreover, at the lowest concentrations tested in HMEC-1 and PHEC cells, as well as the tri-culture, pro-inflammatory gene expression appeared to be induced in the absence of effects on HO-1 expression. Thus, our results indicate that the role of oxidative stress in regulation of DEP-induced pro-inflammatory responses could be a high-dose phenomenon. By contrast, DEP- and DEP-EOM-induced gene expression at the lowest concentrations tested appeared to be triggered through receptor-mediated effects in absence of oxidative stress. While our present data clearly are insufficient to conclude on this matter, they highlight the importance of exploring the role of redox-responses and oxidative stress at exposure-relevant DEP-concentrations [36].

## Conclusion

This study shows that exposure-relevant concentrations of DEP (from 0.12 μg/cm^2^) on the epithelial side of a 3D tri-culture, mimicking the alveolar-capillary barrier, induced increased expression of pro-inflammatory and AhR-regulated genes in the basolateral endothelial cells. These effects were most likely due to soluble organic constituents detached from DEP. Furthermore, direct exposure of HMEC-1 and PHEC to lipophilic organic extracts of DEP induced a comparable up-regulation of pro-inflammatory and AhR-regulated genes, most notably at low concentrations in PHEC (0.15 μg/cm^2^). These effects appeared to be linked to AhR and PAR-2 signaling, and at higher concentrations also involved redox-regulated responses. Thus AhR agonists and other lipophilic constituents appear to be the main drivers of these effects. Although further studies will be necessary to validate these findings, we suggest that lipophilic organic compounds from DEP may cross over the alveolar epithelium triggering inflammatory reactions in remote endothelial cells.

## Methods

### Chemicals

Benzo[*a*]pyrene (B[*a*]P), dimethyl sulfoxide (DMSO) and hydrocortisone were purchased from Sigma-Aldrich (St. Louis, MO). All organic solvents were of > 99% purity (GC or LC-MS grade) and purchased from VWR (Radnor, PA, USA). Analytical standards were obtained from either Fisher Scientific (Hampton, NH, USA) or Sigma-Aldrich (St. Louis, MO, USA). Phorbol-12-myristate-13-acetate (PMA) was purchased from Merck KGaA (Darmstadt, Germany); L-Glutamine (200 mM) from Thermo Fischer Scientific (Scotland); endothelial growth factor from Nerliens Meszansky (Oslo, Norway); penicillin and streptomycin and EC growth medium (EGM-2MV) from Lonza (Walkersville, MD, USA); and MCDB 131, RPMI-1640 and DMEM medium with Glutamax was provided by Life TechnologiesTechnologies (NY, USA); fetal calf serum (FCS) from Biochrom AG (Berlin, Germany). The suspensions of silica nanoparticles with nominal size of 10 nm (SiNP) was purchased from Kisker Biotech (Steinfurt, Germany).

RNA isolation done with RNeasy from Qiagen (Qiagen, Germantown, MD) or NucleoSpin RNA Plus (Macherey-Nagel; Düren, Germany). All real-time Real Time/quantitative-PCR (q-PCR) reagents and TaqMan probes/primers were purchased from Applied Biosystems (Foster City, CA, USA). Cytokine ELISA assays for IL-6 (Human IL-6 CytoSet) and CXCL8 (Human IL-8 CytoSet) were purchased from Biosource International (Camarillo, CA, USA). ELISA assays for MMP-1 were purchased from R&D systems (Minneapolis MN, USA). Cell culture flasks were obtained from Nunc A/S (Roskilde, Denmark) and 12-well plates from Corning, Lowell (MA, USA). The Falcon transwell inserts and additional 6-well TC-Treated Polystyren plate Companion were purchased from Corning (surface area of 4.2 cm^2^; 1 μm pore size; high pore density PET membranes; BD Biosciences, Basel, Switzerland).

### Diesel exhaust particles, chemical extraction and analysis

DEP currently used were collected from the tail-pipe of a diesel engine (Deutz, 4 cylinder, 2.2 l, 500 rpm) running on gas oil,characterized as described elsewhere [40, 41], and kindly provided by Flemming R. Cassee (RIVM). These particles contain approximately 60% OC, corresponding to other OC-rich DEP [73, 74]. Although the current DEP is not necessarily representative of DEP from modern cars, the PAH-composition of these particles resembles what has been reported from other DEPs, with high levels of phenanthrene, fluoranthene, pyrene and chrysene [41, 42]. The detailed approach to characterization of extracts is provided below.

Extraction: DEP extraction was performed with a series of solvents ranging from non-polar to polar using a pressurized extraction system as previously described [75]. The DEP-EOM (10 mg) was extracted separately by a solvent sequence of increasing polarity, from n-hexane to water. Organic solvents were employed using a dynamic mode at constant flow of 0.5 mL/min for 30 min through the extraction vessel. The final water fraction was obtained using first a static mode, where solvent remained in contact with the DEP sample for 5 min, followed by a dynamic extract collection at a flow rate of 0.6 mL/min for 5 min (i.e., a flushing volume of 3.0 mL, more than three internal extraction vessel volumes). Each DEP-EOM fraction was analyzed as described below:

Chemical Analysis: The OC in extracts and the original PM was determined using a thermal optical analyzer (Sunset Laboratories, Tigard, OR, USA). The temperature program began with five steps under an inert helium starting at 300 °C, followed by 500 °C, 600 °C, 700 °C each step for 75 s, and 870 °C for 120 s. Then, the instrument was cooled down to 550 °C and helium with 5% oxygen was introduced with the temperature program starting with 550 °C for 45 s, 625 °C for 45 s, 700 °C for 45 s, and 890 °C for 120 s. For OC analysis 50–150 μg of PM was placed onto pre-baked quartz filter (600 °C, overnight) using a glass rod. DEP-EOM fractions were analyzed by introducing an aliquot (10–80 μL) on the pre-baked quartz filter placed on a heating plate. The solvent was then evaporated at 45 °C for 4–8 min, depending on the type of solvent [73]. To distinguish pyrolyzed OC from EC, laser transmittance at 658 nm was used. As expected no EC was found in the extracts.

The gas chromatography mass spectrometry (GC-MS) revealed only alkanes, PAHs and PAHs derivatives in the extracts. The corresponding aliquots in organic solvents were spiked with deuterated recovery standards (naphthalene-d8, pyrene-d10, and 1-hydroxypyrene-d9) and concentrated to 200 μL under a gentle stream of nitrogen. Water aliquots were also spiked with recovery standards, but concentrated to 200 μL using a vacuum rotary evaporator (7–20 × 10-3 bar, 30 °C). Half of the concentrated sample (100 μL) was then spiked with an internal standard (fluoranthene-d10) and analyzed directly using a gas chromatograph coupled to mass spectrometer (GC-MS). To determine hydroxy-PAHs, the other half of the concentrated sample (100 μL) was evaporated to dryness under gentle stream of nitrogen and mixed with 50 μL of sialylation agent, BSTFA. The mixture was then heated for 10 h at 70 °C, mixed with 50 μL of dichloromethane, and with fluoranthene-d10.

The GC-MS used was a 6890 Series II Plus GC coupled to a 5975C MS detector (Agilent, Santa Clara, CA). Separations were carried out using a 22 m-long DB-5MS column with 0.25 mm internal diameter and 0.25 mm film thickness (J&W Scientific, Rancho Cordova, CA, USA) at a constant helium flow rate of 1.0 mL/min. Samples (1.0 μL) were injected in a splitless mode for 0.5 min at 250 °C. The temperature program started at 35 °C that was held for 2 min, followed by an increase to 140 °C with a 15 °C/min temperature gradient. The last step was an increase to 320 °C with a 10 °C/min temperature gradient, held for 10 min. The total run time was 37 min. The transfer line temperature was set to 280 °C. The MS data were acquired in the full scan mass range of 43–500 m/z using an electron ionization (70 eV). Quantifications were done using eight-point calibrations with the corresponding standard quantification ions listed in Additional file 1: Table S1. For compounds for which standard were not available the nearest isomeric standard was employed.

### Particle size and distribution

The dynamic size measurements were performed at 37 °C in the culture media used in the study, at a concentration of 50 μg/ml. Each particle solution was measured 3 times on a zeta-sizer NANO ZSP (Malvern Instruments Ltd., WR14 1XZ, UK). The results are presented as mean size distribution by intensity. The DEP had a bimodal distribution, with a minor peak around 100 nm and the main peak around 300 nm. As nucleation mode DEP typically is less than 40-50 nm in diameter [15], it seems likely that both peaks could represent agglomerated particles. Interestingly, the 10 nm SiNPs displayed a comparable distribution, peaking around 200-300 nm, suggesting that also these particles primarily occurred as agglomerates when suspended in media (Additional file 1: Figure S6).

### Cell cultures

A 3D tri-culture consisting of three different cell-types, EA-hy926, A549 and PMA-differentiated THP-1 cells, were prepared principally as described by Klein and coworkers [38]. The cells were obtained from the American Type Culture Collection (Manassas, VA, USA). EA.hy 926, A549 and THP-1 cells were maintained in either DMEM with Glutamax, 10% FCS and 1% Hepes (Ea.hy 926 and A549) or in RPMI-1640 with 10% FCS (THP-1) in T75 flasks in a humidified atmosphere at 37 °C with 5% CO_2_, with refreshment of medium twice a week.

Building the 3D tri-cultures started by seeding EA.hy 926 EC on the inverted trans-well inserts at a density of 2.57 × 10^5^ cells/cm^2^. Four h after seeding, the plate with the trans-well inserts was turned back to its original orientation and A549 cells were seeded inside the trans-well (1.28 × 10^5^ cells/cm^2^). Epithelial and endothelial cells were then grown for 3 days at 37 °C and 5% in a humidified incubator with 2 mL of DMEM with Glutamax, 10% FCS and 1% Hepes in the upper and lower chamber; then for 1 day with co-culture media (DMEM with Glutamax and 15% RPMI-1640, 10% FCS and 1% Hepes).On day 3, THP-1 cells were differentiated into macrophage-like cells with PMA (20 ng/mL; PMA-differentiated THP-1 cells). On day 4, differentiated THP-1 cells (2.57 × 10^5^ cells/cm^2^) were added to the inserts and the complete tri-culture was kept in co-culture media with 1% FCS. On day 5 the 3D tri-cultures were ready for exposures.

*Ea. Hy 926 monocultures* were seeded on 6 well plates at a cell density of 250.000 cells/well in 1.5 ml of DMEM with Glutamax, 10% FCS and 1% Hepes 2 days before exposure.

*Human micro-vascular endothelial cells (HMEC-1)*, obtained from Laboratory of the Government Chemist (LGC Standards, Germany), were routinely maintained in MDCB131 medium containing epidermal growth factor (10 ng/mL), hydrocortisone (0.2 μg/mL), penicillin (50 unit/mL), and streptomycin (50 μg/mL) and supplemented with 10% fetal calf serum (FCS), according to the providers instructions.

*Primary human endothelial cells (PHEC)* were isolated from adipose tissue obtained from liposuction material from abdominal regions of four healthy female donors (aged 22–35 years; BMI: 23–30) undergoing cosmetic surgery [76]. The stromal vascular fraction was isolated as described previously [76]. Briefly, lipo-aspirates were washed and digested using 0.1% collagenase A type 1. After centrifugation, the cell pellet was filtered through 100 μm and then 40 μm cell sieves. Cells were obtained from the interface after Lymphoprep gradient separation (Axis Shield; Oslo, Norway). CD44+ cells were removed using Dynabeads (Dynabeads Pan Mouse IgG; Invitrogen Dynal AS, Oslo, Norway) according to the manufacturer’s description. PHEC were plated at 2 × 10^6^ cells per 75-cm^2^ tissue culture flask Nunc A/S (Roskilde, Denmark). Cells were maintained at 37 °C in an atmosphere of 5% CO_2_ in humid air using endothelial cell growth medium (EGM-2MV) with supplements according to the manufacturer’s description; human AB-serum (serum from individuals with blood-type AB) was used instead of FCS. Cells were routinely passaged every 3–4 days.

### In vitro exposures

*3D tri-culture:* prior to exposure, the media was changed to co-culture media without FCS. DEP suspended in co-culture media without FCS were added to the upper chamber. After 2 or 20 h exposure, cells from the apical compartment (A549 and PMA-differentiated THP-1 cells) and the basolateral compartment (EAhy.926 endothelial cells) were harvested and mRNA was isolated using the RNeasy mini kit according to the protocol from the manufacturer (Qiagen, Germantown, MD). In separate experiments the tri-culture and EAhy.926 endothelial cells were exposed to Si10 in absence of FCS for 3 and 6 h prior to harvesting of mRNA.

*HMEC-1* and *PHEC* were grown to near-confluency and serum-starved for a minimum of 12 h prior to exposure. Cells were then exposed by removing the media and adding growth medium without FCS containing various DEP-EOM suspended in DMSO or DMSO alone. After 2, 5 or 24 h exposure, growth-medium was obtained for ELISA, cells were harvested and mRNA extracted. In all experiments that included chemical inhibitors, cells were pre-treated for 30 min with the inhibitor, then exposed to the DEP-EOM.

Chemicals were commonly prepared as stock solution in DMSO. The final concentration of solvent did not exceed 0.2% (*v*/v); control cultures received similar concentration of DMSO. Stock solution of Si10 was dispersed in sterile water (2.3 mg/ml) and sonicated for approximately 2 min on ice (until specific ultrasound energy of 420 J was given to the nanoparticles). Bovine serum albumin (BSA, final concentration 0.15%) and phosphate buffered saline (PBS, final dilution 1×) were then added to the particle solution, according to the method by Bihari and co-workers [77].

### Gene expression analysis by real-time RT-PCR

RNA was isolated using NucleoSpin RNA Plus (Macherey-Nagel; Düren, Germany) or RNeasy from Qiagen (Qiagen, Germantown, MD), and reverse transcribed to cDNA on a PCR System 2400 (PerkinElmer, Waltham, MA, USA) using a High Capacity cDNA Archive Kit (Applied Biosystems, Foster City, CA, USA). Real-time PCR was performed using pre-designed TaqMan Gene Expression Assays and TaqMan Universal PCR Master Mix and run on Applied Biosystems 7500 fast software. Gene expression of induced IL-1α (Hs00174092_m1), IL-1β (Hs01555410_m1), IL-6 (Hs00174131_m1), CXCL8 (Hs00174103_m1), COX-2 (Hs00153133_m1), MMP-1 (Hs00899658_m1), HO-1(Hs01110250_m1), PAI-2/SERPINB2 (Hs01010736_m1), CYP1A1 (Hs00153120_m1) and Cyp1B1 (Hs02382916_s1) were normalized against GAPDH (Hs02758991_g1) and expressed as fold change compared to untreated control as calculated by the ΔΔCt method (ΔCt = Ct[Gene of Interest] – Ct[18S]; ΔΔCt = ΔCt[Treated] – ΔCt[Control]; Fold change = 2[-ΔΔCt]).

### ELISA

The amount of MMP-1, IL-6 and CXCL8 in cell medium was measured by ELISA according to the manufacturers’ guideline. An increase in color intensity was quantified by a plate reader (TECAN Sunrise, Phoenix Research Products, Hayward, CA, USA) equipped with a dedicated software (Magellan V I.10; Tecan Austria GmbH, Grödig-Salzburg, Austria).

### Flow cytometry

Flow cytometry was performed for determination of cell surface antigen expression of PHEC from one donor as described previously [46]. Cells were analyzed using a Gallios flow cytometer from Beckman Coulter with Gallios software.

### Statistical analysis

Statistical analysis was performed by ANOVA with Holm-Sidak post-test for multiple comparisons. As ANOVA cannot be performed on normalized data, the gene expression data were analyzed using the deltaCT-values from the q-PCR measurements. All calculations were performed using GraphPad Prism 7 software (GraphPad Software, Inc., San Diego, CA).

## Additional file

Additional file 1: **Figure S1.** In a 3D tri-culture, exposure to SiNP on the epithelial side, induced COX-2 on the epithelial side, but not in the endothelial cells. Furthermore EAhy.926 endothelial cells exposed directly to SiNP up-regulated COX-2. **Figure S2.** The amount of volatile/semi-volatile compounds extracted decreased according to polarity of the solvents. **Figure S3.** Cytotoxicity of DEP-EOM in HMEC-1 and PHEC. **Figure S4.** Lipophilic DEP-EOMs cause CXCL8 secretion in HMEC-1 cells. **Figure S5.** PHEC were 99% CD31-positive. **Figure S6** Size distribution, DEP and SiNP. **Table S1.** GC-MS quantified compounds with corresponding MS ions and calibration standards. (DOCX 2036 kb)

## Abbreviations

AhR: Aryl hydrocarbon receptor
B[a]P: Benzo[*a*]pyrene
CH223191: AhR-inhibitor
CVD: Cardiovascular disease
CYP: Cytochrome P450
DCM-EOM: Organic material of DEP extracted by: Dichloromethane
DEP: Diesel exhaust particles
DEP-EOM: Extractable organic material of DEP
DMSO: Dimethyl sulfoxide
ENMD-1068: PAR-2-inhibitor
HMEC-1: Human microvascular endothelial cells
HO-1: Heme oxygenase-1
IL: Interleukin
Methanol-EOM: Organic material of DEP extracted by: Methanol
MMP-1: Matrix metalloproteinase-1
MW: Molecular weight
NAC: N-acetylcysteine
NF-κB: Nuclear factor-κB
*n*-Hex-EOM: Organic material of DEP extracted by: *n*-hexane
OC: Organic chemicals
PAHs: Polycyclic aromatic hydrocarbons
PAI-2: Plasminogen activator inhibitor 2
PAR-2: Protease-activated receptor-2
PHEC: Primary human endothelial cells
PM: Particulate matter
PM_2.5_: Fine traffic-derived PM
PMA: Phorbol-12-myristate-13-acetate
ROS: Reactive oxygen species
SiNP: SiO_2_ nanoparticles
Water-EOM: Organic material of DEP extracted by: Water at 25 °C
XREs: AhR-response elements
