# Supplementary material for: Lipophilic components of diesel exhaust particles induce pro-inflammatory responses in human endothelial cells through AhR dependent pathway(s)
Source: Part Fibre Toxicol. 2018 May 11;15:21. doi: 10.1186/s12989-018-0257-1 (PMC5948689; doi:10.1186/s12989-018-0257-1)
Supplement: Supplementary file 1 — Figure S1. In a 3D tri-culture, exposure to SiNP on the epithelial side, induced COX-2 on the epithelial side, but not in the endothelial cells. Furthermore EAhy.926 endothelial cells exposed directly to SiNP up-regulated COX-2. Figure S2. The amount of volatile/semi-volatile compounds extracted decreased according to polarity of the solvents. Figure S3. Cytotoxicity of DEP-EOM in HMEC-1 and PHEC. Figure S4. Lipophilic DEP-EOMs cause CXCL8 secretion in HMEC-1 cells. Figure S5. PHEC were 99% CD31-positive. Figure S6 Size distribution, DEP and SiNP. Table S1. GC-MS quantified compounds with corresponding MS ions and calibration standards. (DOCX 2036 kb) [file 12989_2018_257_MOESM1_ESM.docx]

# Additional file 1

FIGURE S1**: In a 3D tri-culture, exposure to SiNP on the epithelial side, induced COX-2 on the epithelial side, but not in the endothelial cells. Furthermore EAhy.926 endothelial cells exposed directly to SiNP up-regulated COX-2.** SiNP (12.5 or 25 μg/mL) were applied to the epithelial side of the 3D tri-culture (A). EA.hy926 cells in monoculture were directly exposed to the same concentrations of SiNP (B). After 3 and 6 h of exposure, alveolar and endothelial cells were harvested and the expression of COX-2 was measured by q-PCR. The mRNA levels are relative to the untreated control. The results are expressed as mean ± SEM (A/B: n=2/3). *Statistically significant difference from unexposed controls.


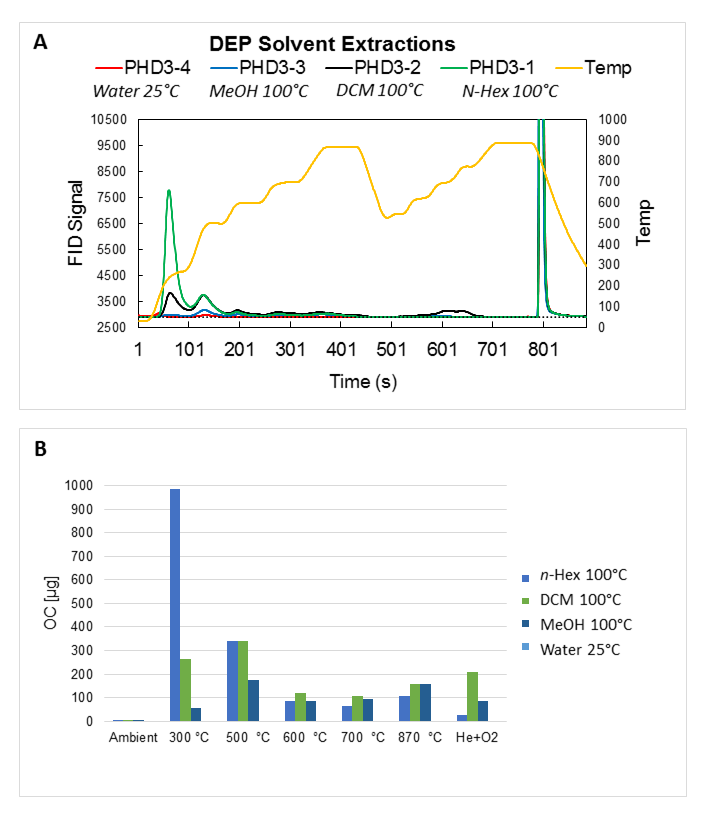


FIGURE S2**: The amount of volatile/semi-volatile compounds extracted decreased according to polarity of the solvents.** Organic carbon speciation using thermal optical analyzer (TOA) for different polarity solvent fractions obtained by DEP extraction shown as a) overlaid thermograms and b) processed data (ratios of peak areas converted to µg). The temperatures shown are the steps used for thermal desorption and pyrolytic evolution of carbonaceous species within TOA analysis. The results are based on EOM from 10 mg of the original DEP.

******


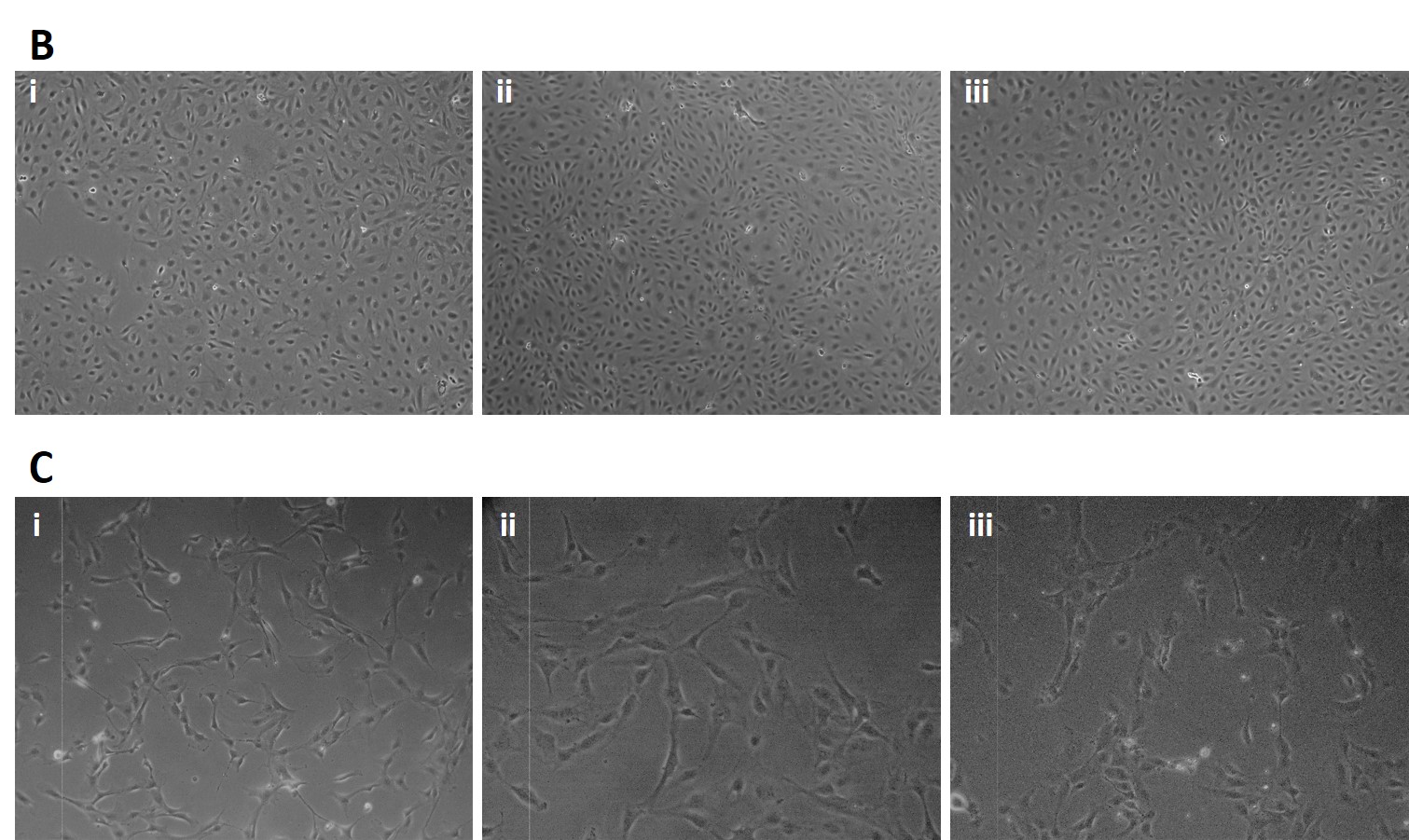


FIGURE S3: **Cytotoxicity of DEP-EOM in HMEC-1 and PHEC.** Cytotoxicity in HMEC-1 cells was screened by WST-1 assay for effects on cell proliferation (A). Cells seeded in a 96-well plate were exposed to 200 μL of growth medium containing DEP-EOM at concentrations corresponding to 0, 2, 5, 10, 50 and 100 μg/mL of native particles, these concentrations equal 0.9, 2.3, 4.7, 23.5 and 47.0 μg/cm^2^ (A; n=1). Furthermore, cytotoxicity was also examined visually by light microscopy in all experiments with PHECs and HMEC-1 (B and C). No visual cytotoxicity was observed in PHEC pre-exposure (i), exposed 24 h to DMSO (ii) and 0.75 μg/cm^2^ n-hexane (iii) (B). No visual cytotoxicity was observed in HMEC-1 pre-exposure (i), exposed to 24 h DMSO (ii) and 7.5 μg/cm^2^ n-hexane (iii) (C).


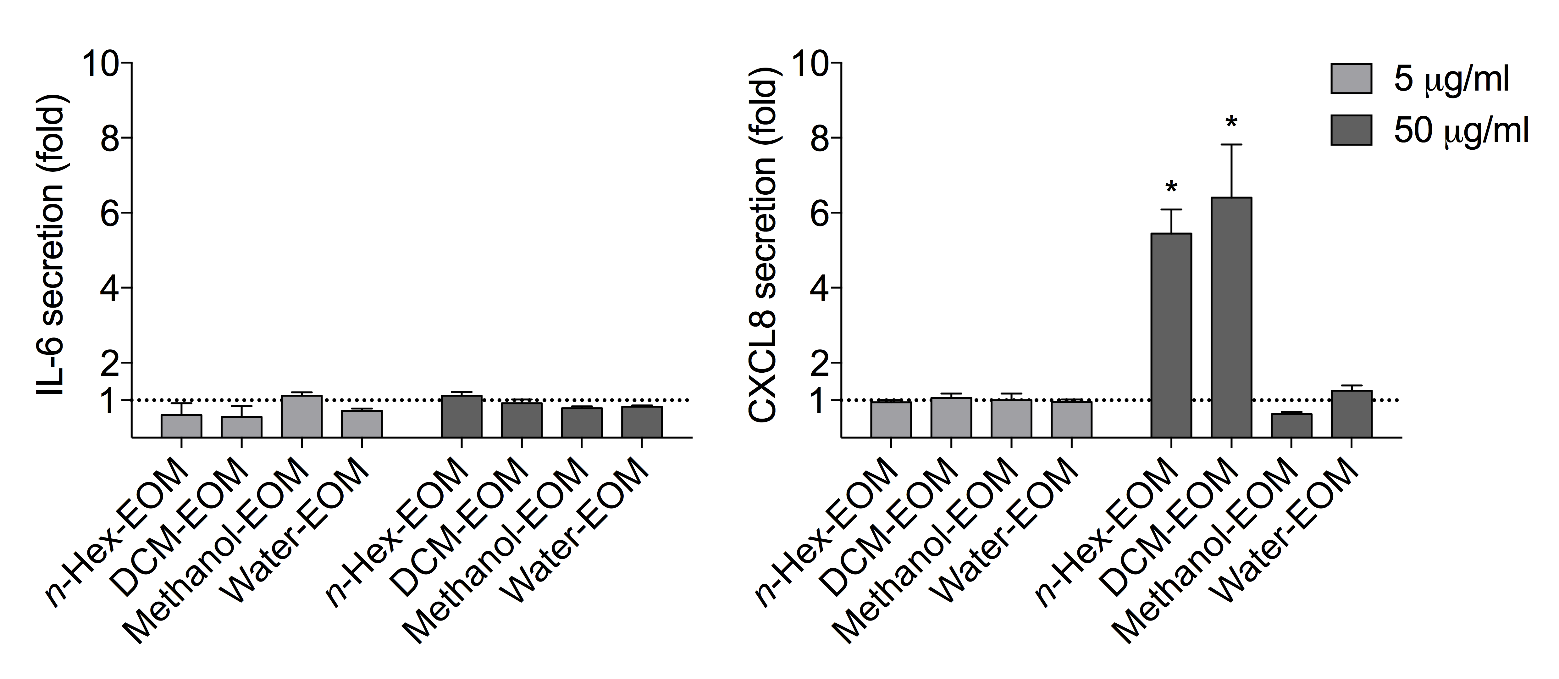


FIGURE S4. **Lipophilic DEP-EOMs cause CXCL8 secretion in HMEC-1 cells.** Cells were exposed to DEP-EOM at concentrations corresponding to 5 and 50 μg/mL (0.75 and 7.5 μg/cm^2^) of native particles, or vehicle (DMSO) alone for 24 h. IL-6- and CXCL8-levels in the medium were measured by ELISA. The protein levels are relative to the DMSO, represented by the dotted line at 1. The results are expressed as mean ± SEM (n≥ 3). *Statistically significant difference from unexposed controls.


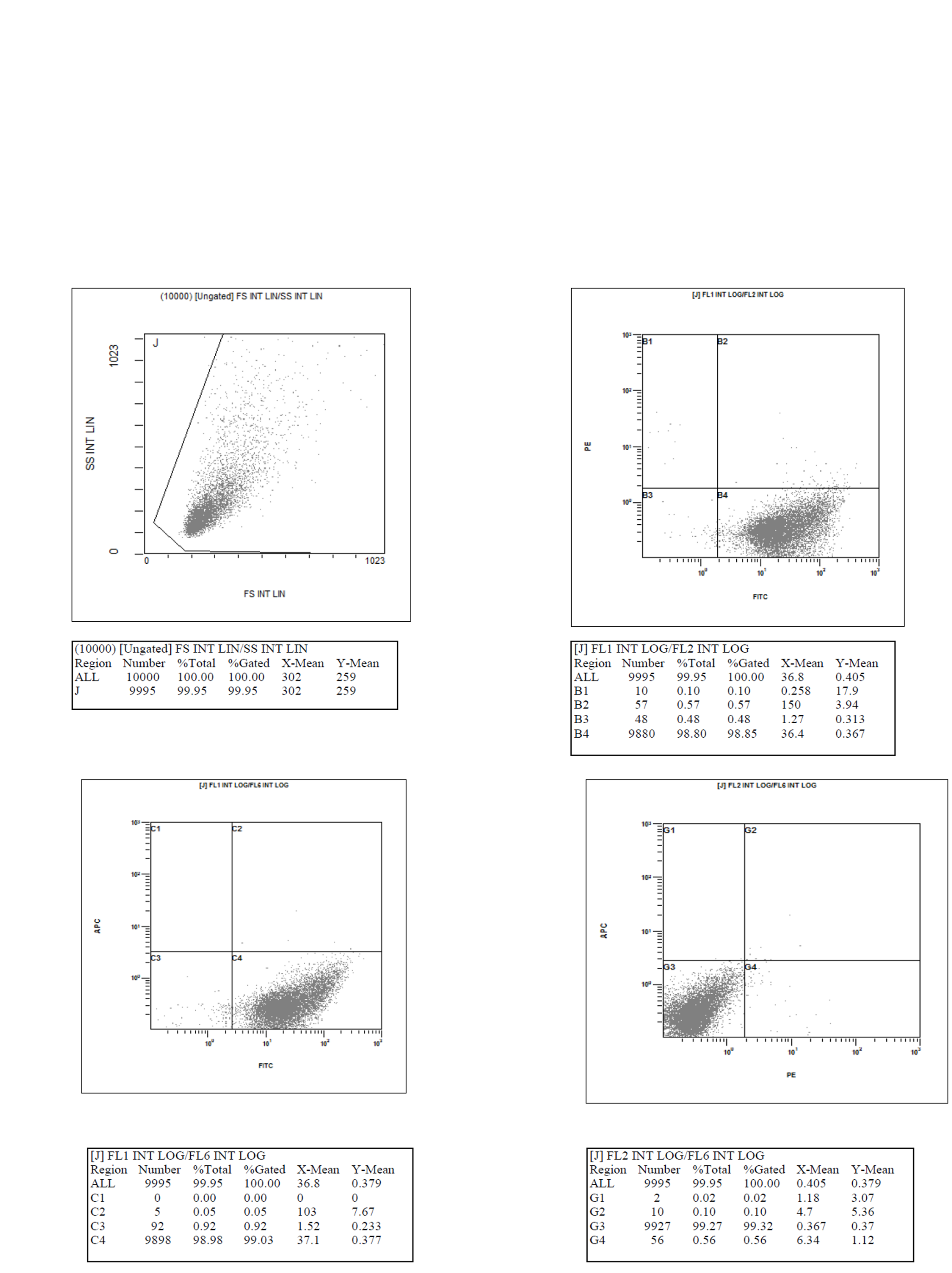


FIGURE S5: **PHEC were 99% CD31-positive.** PHEC from one donor were stained with FITC fluorescent antibody specific for the endothelial cell marker CD31. The gated cells were 99% CD31-positive, indicating that the PHEC cultures were 99% pure. The isolation and cultivation of these cells has been described elsewhere (Szoke et al. 2012).


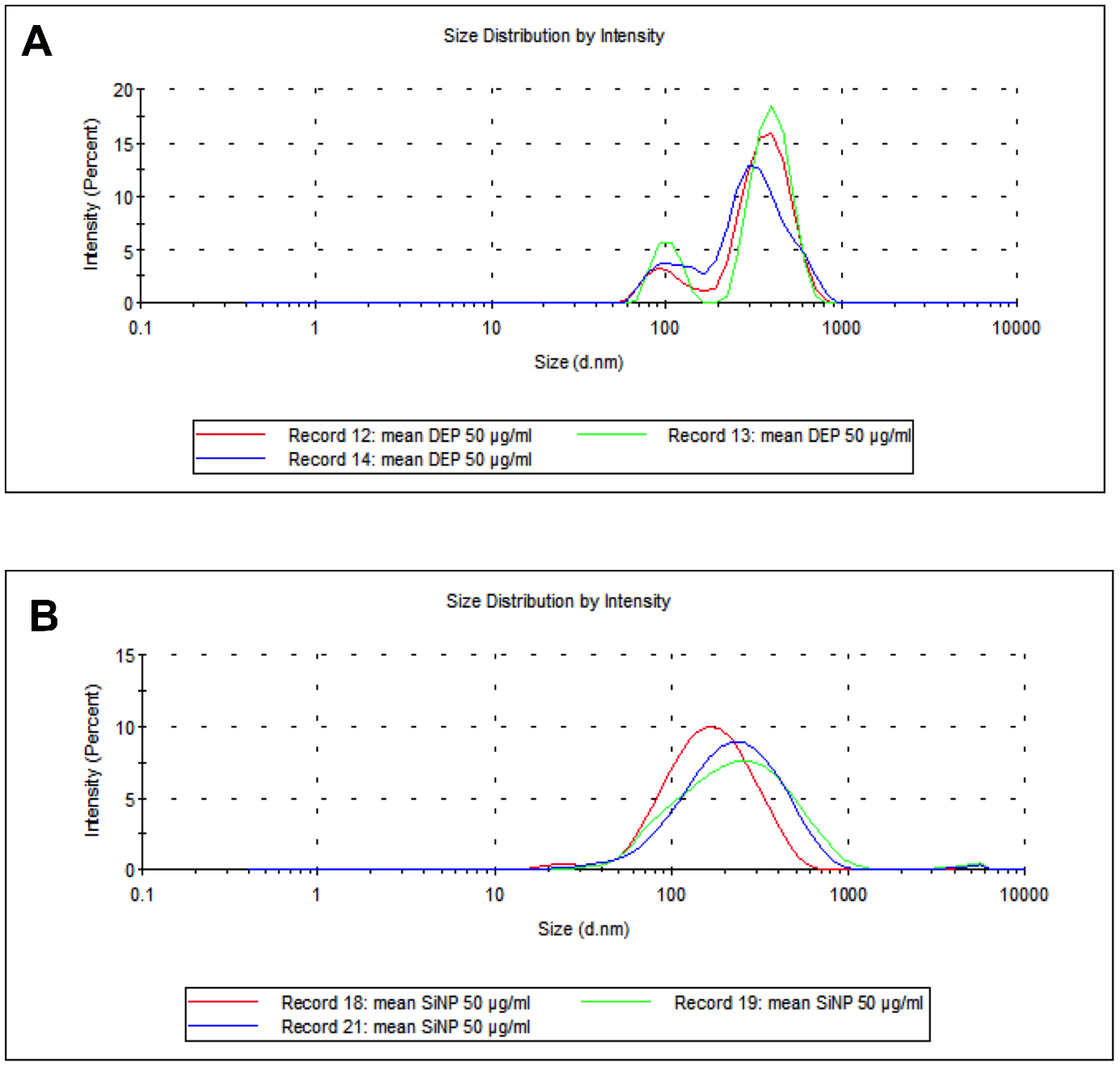


FIGURE S6: **Size distribution, DEP and SiNP.** The dynamic size measurements were performed at 37°C in the culture media used in the study, at a concentration of 50 µg/ml. Each particle solution was measured 3 times on a zeta-sizer NANO ZSP (Malvern Instruments Ltd, WR14 1XZ, UK). The results are presented as mean size distribution by intensity.

**Table S1:** GC-MS quantified compounds with corresponding MS ions and calibration standards.

| Group | Compound | Formula | Quantification Ion *m/z* | Confirmation Ions *m/z* | | Calibration Standard |
| --- | --- | --- | --- | --- | --- | --- |
| PAHs | phenanthrene | C_14_H_10_ | 178 | 152 | 89 | phenanthrene |
|  | anthracene | C_14_H_10_ | 178 | 152 | 89 | anthracene |
|  | methyl-phenanthrene/anthracene  (A–E) | C_15_H_12_ | 192 | 191 | 165 | 2-methylanthracene |
|  | fluoranthene | C_16_H_10_ | 202 | 106 | 92 | fluoranthene |
|  | pyrene | C_16_H_10_ | 202 | 174 | 101 | pyrene |
|  | methyl-fluoranthene/pyrene  (A–G) | C_17_H_12_ | 216 | 215 | 190 | 1-methylpyrene |
|  | benzo[a]anthracene | C_18_H_12_ | 228 | 114 | 101 | chrysene |
|  | chrysene | C_18_H_12_ | 228 | 114 | 101 | chrysene |
|  | benzo[b]fluoranthene | C_20_H_12_ | 252 | 126 | 113 | benzo[b]fluoranthene |
|  | benzo[a]pyrene | C_20_H_12_ | 252 | 126 | 113 | benzo[a]pyrene |
| Oxy-PAHs | 9-fluorenone | C_13_H_8_O | 180 | 152 | 126 | 9-fluorenone |
|  | xanthone | C_13_H_8_O_2_ | 196 | 168 | 139 | xanthone |
|  | anthrone (A–E) | C_14_H_10_O | 194 | 165 | 139 | anthrone |
| Hydroxy-PAHs | 2-hydroxybiphenyl | C_12_H_10_O | 242 | 227 | 211 | 2-hydroxybiphenyl |
|  | 3-hydroxybiphenyl | C_12_H_10_O | 242 | 227 | 211 | 4-hydroxybiphenyl |
|  | 4-hydroxybiphenyl | C_12_H_10_O | 242 | 227 | 211 | 4-hydroxybiphenyl |
|  | 2-hydroxy-9-fluorenone | C_13_H_8_O_2_ | 268 | 195 |  | 2-hydroxy-9-fluorenone |
|  | 9-phenanthrol | C_14_H_10_O | 266 | 251 | 235 | 9-phenanthrol |
|  | 1-hydroxypyrene | C_16_H_10_O | 290 | 275 | 250 | 1-hydroxypyrene |
| Nitro-PAHs | 1-nitropyrene | C_16_H_9_NO_2_ | 247 | 231 | 215 | 1-nitropyrene |
| Alkanes | tetradecane | C_14_H_30_ | 57 | 85 | 198 | tetradecane |
|  | pentadecane | C_15_H_32_ | 57 | 85 | 212 | tetradecane |
|  | hexadecane | C_16_H_34_ | 57 | 85 | 226 | tetradecane |
|  | heptadecane | C_17_H_36_ | 57 | 85 | 240 | tetradecane |
|  | octadecane | C_18_H_38_ | 57 | 85 | 254 | tetradecane |
|  | nonadecane | C_19_H_40_ | 57 | 85 | 268 | tetradecane |
|  | eicosane | C_20_H_42_ | 57 | 85 | 282 | eicosane |
|  | henicosane | C_21_H_44_ | 57 | 85 | 296 | eicosane |
|  | docosane | C_22_H_46_ | 57 | 85 | 310 | eicosane |
|  | tricosane | C_23_H_48_ | 57 | 85 | 324 | eicosane |
|  | tetracosane | C_24_H_50_ | 57 | 85 | 338 | eicosane |
|  | pentacosane | C_25_H_52_ | 57 | 85 | 352 | eicosane |
|  | hexacosane | C_26_H_54_ | 57 | 85 | 366 | eicosane |
|  | heptacosane | C_27_H_56_ | 57 | 85 | 380 | eicosane |
|  | octacosane | C_28_H_58_ | 57 | 85 | 394 | eicosane |
